# Improvement in disease activity among patients with rheumatoid arthritis who switched from intravenous infliximab to intravenous golimumab in the ACR RISE registry

**DOI:** 10.1007/s10067-022-06116-z

**Published:** 2022-03-21

**Authors:** John Tesser, Iris Lin, Natalie J. Shiff, Soumya D. Chakravarty, Gabriela Schmajuk, Nevin Hammam, Sheetal Desai

**Affiliations:** 1 Arizona Arthritis & Rheumatology Associates, 4550 E. Bell Road, Suite 172, Phoenix, AZ 85032 USA; 2 Janssen Scientific Affairs, LLC, Horsham, PA USA; 3 Adjunct, Department of Community Health and Epidemiology, University of Saskatchewan, Saskatoon, SK Canada; 4 Drexel University College of Medicine, Philadelphia, PA USA; 5 University of California San Francisco, San Francisco, CA USA; 6 University of California, Irvine, Irvine, CA USA

**Keywords:** Rheumatoid arthritis, Intravenous golimumab, Intravenous infliximab, Tumor necrosis factor, Disease activity, Registry

## Abstract

Infliximab and golimumab are intravenously (IV) administered tumor necrosis factor inhibitors approved to treat moderate-to-severe rheumatoid arthritis (RA) with concomitant methotrexate. Owing to differences in biologic construct, patients with IV-infliximab treatment failure may benefit from switching to IV-golimumab. Utilizing the ACR’s Rheumatology Informatics System for Effectiveness (RISE), a large electronic health records registry based in the USA, we assessed RA disease activity in patients switching from IV-infliximab to IV-golimumab. This retrospective, longitudinal, single-arm study included adults (≥ 18 years) with ≥ 1 RA diagnosis code between 2014 and 2018 and ≥ 1 IV-infliximab prescription within 6 months of a new IV-golimumab order (index date). Longitudinal assessments of disease activity using the Clinical Disease Activity Index (CDAI) were calculated in patients continuing IV-golimumab for 6–9- and 9–12-months post-switch. Paired *t*-tests evaluated significance of mean improvements during the follow-up periods. Most RA patients with disease activity assessments during the 6-month follow-up (*N* = 100; mean age: 65.3 years; 81% female; 74% white) demonstrated moderate-to-high disease activity (CDAI: 73% [38/52]) at enrollment. On average, patients showed significant improvement in disease activity within 6–9 months of switching; mean CDAI scores improved from 21.3 to 14.1 (*p* < 0.0001) and were durable through 9–12 months of treatment. Real-world patients with moderate-to-high disease activity who switched from IV-infliximab to IV-golimumab demonstrated significant and sustained improvements post-switch as measured by the CDAI.

**Table:** 

**Key Points** • *This study used real-world data from the Rheumatology Informatics System for Effectiveness (RISE) registry to evaluate the efficacy of directly switching from intravenous (IV)-infliximab to IV-golimumab to control rheumatoid arthritis (RA) disease activity.* • *Most IV-infliximab patients had moderate-to-high disease activity at the time of the switch.* • *On average, IV-golimumab was effective in improving RA disease activity after switching from IV-infliximab as measured by the Clinical Disease Activity Index.* • *These data suggest that real-world RA patients with persistent symptoms despite treatment with IV-infliximab may realize improved disease control with a switch to IV-golimumab.*

**Supplementary Information:**

The online version contains supplementary material available at 10.1007/s10067-022-06116-z.

## Introduction

Golimumab, a fully human monoclonal antibody administered intravenously (IV) or subcutaneously [1, 2], and IV-infliximab, a chimeric monoclonal antibody [3], are tumor necrosis factor inhibitors (TNFi). TNFi are biologic disease-modifying anti-rheumatic drugs (bDMARDs) approved to treat moderate-to-severe rheumatoid arthritis (RA). IV-infliximab and IV-golimumab are both approved for use in combination with methotrexate (MTX). IV-golimumab and IV-infliximab safety and efficacy in RA patients are well characterized across multiple clinical trials [4–9]. Individual clinical trial data, though not from a head-to-head phase 3 study, suggest similar efficacy with these IV TNFi [4–9]. Conversely, these agents differ with respect to the reported incidence of certain adverse reactions [10, 11]. RA patients commonly switch to a different TNFi after initial course failure. Although data from one randomized controlled trial supporting TNFi switching are acknowledged [12], other data are lacking.

The Rheumatology Informatics System for Effectiveness (RISE) registry is a Health Insurance Portability and Accountability Act-compliant, Qualified Clinical Data Registry developed by the American College of Rheumatology (ACR) to passively collect electronic health record data for clinicians and researchers [13]. Using real-world data from RISE, this study evaluated the efficacy of switching from IV-infliximab to IV-golimumab to control RA disease activity.

## Patients and methods

### Study design and data source

**Fig. 1:**
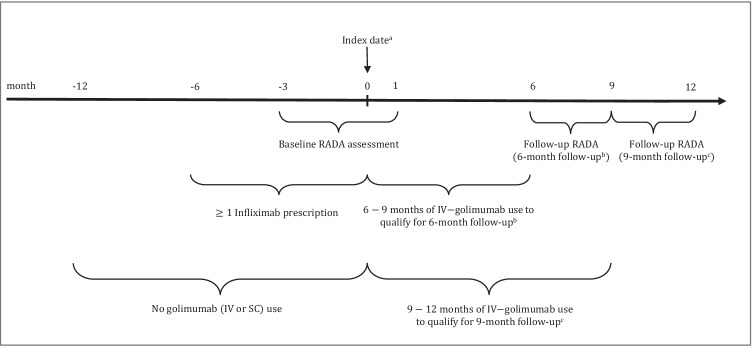
Study design for efficacy evaluation of switching from IV-infliximab to IV-golimumab in controlling RA disease. All medication use was reported from patient medication reconciliation, e-prescription, or procedure (medication administration) tables. ^a^Date IV-golimumab medication first appeared in the patient medication reconciliation, e-prescription, or procedure (medication administration) tables. ^b^Continued IV-golimumab use, with no gaps in treatment, was required for 6–9 months after the index date for the 6-month follow-up. ^c^Continued IV-golimumab use, with no gaps in treatment, was required for 9–12 months after the index date for the 9-month follow-up. IV, intravenous; RA, rheumatoid arthritis; RADA, rheumatoid arthritis disease activity; SC, subcutaneous

This retrospective, longitudinal, single-arm study (Fig. 1) was conducted using data from the RISE registry. RISE contains data collected during routine clinical care, primarily in private rheumatology practices in the United States (US). Data contribution began in January 2014 and included patient demographics, diagnoses, procedures, medications, laboratory test results, RA disease activity scores, and vital signs. In 2018, the database held records from 1113 providers (226 practices accounting for ~ 30% of the US rheumatology clinical workforce) and 1.6 million patients. This study was approved by a central Institutional Review Board (Western IRB) and the University of California at San Francisco (UCSF) IRB.

### Patient population

**Fig. 2:**
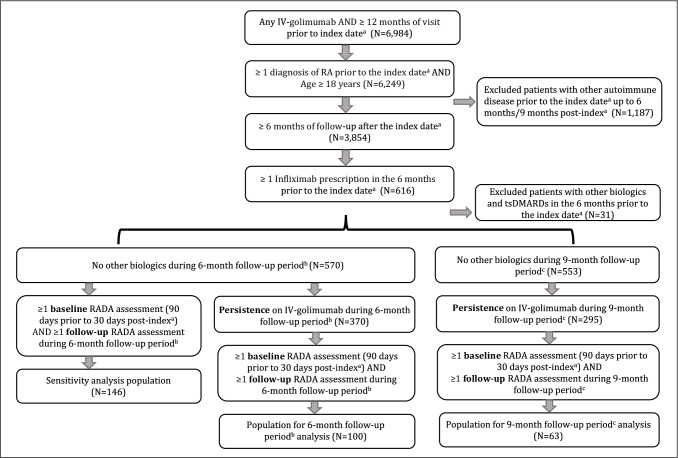
Study patient profile. All medication use was reported from patient medication reconciliation, e-prescription, or procedure (medication administration) tables. ^a^Date IV-golimumab medication first appeared in the patient medication reconciliation, e-prescription, or procedure (medication administration) tables. ^b^Continued IV-golimumab use, with no gaps in treatment, was required for 6–9 months after the index date for the 6-month follow-up. ^c^Continued IV-golimumab use, with no gaps in treatment, was required for 9–12 months after the index date for the 9-month follow-up. IV, intravenous; RA, rheumatoid arthritis; RADA, rheumatoid arthritis disease activity; tsDMARD, targeted synthetic disease-modifying antirheumatic drug

Adults (≥ 18 years of age) in this study had ≥ 1 International Classification of Diseases (ICD) RA diagnosis code (ICD-9-code: 714.*; ICD-10-codes: M05.*, M06.*, excluding M06.4 [inflammatory polyarthropathy]) between 2014 and 2018, ≥ 1 recorded IV-infliximab prescription within 6 months of “new” IV-golimumab use, and a rheumatoid arthritis disease activity (RADA) measure both at baseline and at least once during the follow-up periods (Fig. 2). The index date was defined as the date the medication first appeared in the patient medication reconciliation, e-prescription, or procedure (medication administration) tables (see “7” section, below). The baseline RADA must have been completed between 90 days prior to and up to 30 days after the index date. “New” use was defined as no IV-golimumab within 12 months prior to the index date. Continued IV-golimumab use, with no gaps in treatment, was required for 6–9 months after the index date (6-month follow-up) and for 9–12 months after the index date (9-month follow-up) for patient inclusion in respective analyses. A treatment gap was defined as ≥ 4 weeks between actual and planned IV-golimumab prescription based on every-8-week administration per the approved dosing regimen. A sensitivity analysis of disease activity for the 6-month follow-up included all patients with a RADA at baseline and allowed gaps of ≥ 4 weeks.

This study included all patients with index dates between January 1, 2014, and June 30, 2018, and used data available until December 31, 2018, to allow for sufficient follow-up time. Follow-up time was the period between a patient’s first and most recent face-to-face visits with the rheumatologist. Patients with ≥ 2 ICD codes for systemic lupus erythematosus, psoriatic arthritis, scleroderma, inflammatory bowel disease, or sarcoidosis at any time pre-index date or during the 6- or 9-month follow-up periods were excluded, ensuring study participants were prescribed IV-golimumab to treat RA only. Patients who had recorded use of any other biologics or tofacitinib within 6 months prior to the index date or 6-9 months after the index date for 6-month and 9-month follow-up analyses, respectively, were excluded, as were pregnant women.

#### Sociodemographic variables

Baseline demographics including age, sex, race, ethnicity, geographic regions, insurance type, and rheumatology practice type were assessed within 12 months prior to the index date. Geographic regions were defined according to the US Census.

#### Clinical and laboratory variables

Charlson comorbidity index scores were calculated, based on single ICD-9 and ICD-10 codes within 12 months prior to the index date, according to the Deyo modification [14, 15]; body mass index (BMI) was also reported during this timeframe. Serum C-reactive protein concentration and erythrocyte sedimentation rate within 3 months prior to the index date were reported.

#### Medication exposure

Medication information, including concomitant medication use, was derived from patient reports during face-to-face visits in all practices; e-prescription data sent electronically from provider to pharmacy in ~ 20% of practices; and, in a minority of cases, procedure (administration) codes. The number of IV-infliximab prescriptions within 6 months prior to the index date and the number of days separating the most recent IV-infliximab prescription and the index date were reported. The cumulative dose of IV-infliximab prescribed within 6 months prior to the index date was calculated from the medication administration description field text, where available, by one of two methods:

1 $$infliximab\;dose\left(\frac{mg}{kg}\right)\times patient\;weight\left(kg\right)\times number\;of\;infliximab\;doses$$

2 $$infliximab\;dose\left(\frac{mg}{dose}\right)\times number\;of\;infliximab\;doses$$

Patients were considered to have received a prior biologic or targeted synthetic DMARD if they had recorded prescription of ≥ 1 of the following drugs within 6–12 months prior to the index date: abatacept, adalimumab, certolizumab, etanercept, infliximab, rituximab, tocilizumab, or tofacitinib.

### Outcome measures

RA disease activity was primarily assessed using the Clinical Disease Activity Index (CDAI) at baseline (- 90 to + 30 days of index date) and during the 6- or 9-month follow-up period post-switch to IV-golimumab. For these periods, respectively, any assessment available between 6–9 and 9–12 months after the index date was eligible for analysis. If multiple assessments were available for these periods, those closest to 6-9 months after the index dates were analyzed. The CDAI sums the swollen (0–28) and tender (0–28) joint counts and the rating of global disease activity (0–10) provided by the physician and patient [16]. The Routine Assessment of Patient Index Data 3 (RAPID3), a patient-reported outcome measure without formal joint counts that consists of the Multidimensional Health Assessment Questionnaire patient self-report RA Core Data Set measures for physical function, pain, and patient global estimate (score range: 0–30) [17], was also documented in some settings.

### Statistical analyses

Observed data were summarized with parametric and non-parametric descriptive statistics as appropriate. CDAI scores were reported as mean absolute scores and by categories of disease activity (remission/low/moderate/high) as recommended by ACR [18]. Changes in CDAI scores from baseline were assessed using paired *t*-tests. Shifts in patient distributions across disease activity categories were assessed using one-way repeated measures ANOVA. RAPID3 data were similarly summarized. Statistical testing was two-sided, with a significance level of < 0.05. Analyses were performed using Stata Statistical Software (Release 15; StataCorp, LLC; College Station, TX).

## Results

### Patient disposition and baseline characteristics

Among eligible patients with a baseline and follow-up RADA measure, 100 (52 with CDAI data) and 63 (32 with CDAI data) reported IV-golimumab persistence for ≥ 6 and ≥ 9 months, respectively; patients with and without recorded CDAI data were generally similar (Table 1); sensitivity analyses allowing treatment gaps ≥ 4 weeks included 146 patients (81 with CDAI data; Online Resource 1). Among the 52 patients with CDAI scores at 6-month follow-up, the mean (standard deviation [SD]) age was 64.8 (11.4) years; 81% were female, 79% were white, and 38% were privately insured. On average, patients were followed for 75 months and had 3.3 rheumatology office visits within 12 months prior to the index date; 56% of patients reported receiving ≥ 2 IV-infliximab prescriptions within 6 months prior to the index date (average cumulative dose of 1686 mg among those with data available); and 52% were receiving concomitant MTX (Table 1). Baseline characteristics were consistent across patient cohorts with CDAI vs. RAPID3 data (data not shown) and between the 6- and 9-month follow-up cohorts (Table 1).

**Table 1:** Baseline characteristics of RA patients who switched from IV-infliximab to IV-golimumab with data available for 6-month (6–9 months after switch) and 9-month (9–12 months after switch) follow-up analyses who had any RADA score (CDAI or RAPID3), and with data available for 6-month and 9-month follow-up analyses who had a CDAI score only

Variables		6-month follow-up		9-month follow-up	
		Any RADA (*N* = 100)	CDAI (*N* = 52)	Any RADA (*N* = 63)	CDAI (*N* = 32)
Age (years), mean (SD)		65.3 (11.4)	64.8 (11.4)	67.1 (11.0)	66.9 (10.4)
Female, *n* (%)		81 (81.0)	42 (80.8)	52 (82.5)	26 (81.2)
Race and ethnicity, *n* (%)	White (non-Hispanic)	74 (74.0)	41 (78.8)	44 (69.8)	23 (71.9)
	Asian	4 (4.0)	1 (1.9)	1 (1.6)	0 (0.0)
	Black or African American	3 (3.0)	2 (3.8)	6 (9.5)	4 (12.5)
	Others^a^	7 (7.0)	5 (9.6)	4 (6.3)	2 (6.2)
	Missing	8 (8.0)	0 (0.0)	7 (11.1)	2 (6.2)
	Hispanic or Latino	4 (4.0)	3 (5.8)	1 (1.6)	1 (3.1)
Insurance, *n* (%)	Medicare	42 (42.0)	22 (42.3)	27 (42.9)	16 (50.0)
	Medicaid	8 (8.0)	5 (9.6)	8 (12.7)	7 (21.9)
	Private	37 (37.0)	20 (38.5)	20 (31.8)	7 (21.9)
	Other	2 (2.0)	2 (3.8)	2 (3.2)	1 (3.1)
	Missing	11 (11.0)	3 (5.8)	6 (9.5)	1 (3.1)
US geographic divisions, *n* (%)	New England	0 (0.0)	0 (0.0)	0 (0.0)	0 (0.0)
	Mid-Atlantic	6 (6.0)	2 (3.8)	4 (6.4)	2 (6.2)
	East North Central	25 (25.0)	17 (32.7)	16 (25.4)	12 (37.5)
	West North Central	5 (5.0)	1 (1.9)	5 (7.9)	0 (0.0)
	South Atlantic	34 (34.0)	15 (28.8)	26 (41.3)	9 (28.1)
	East South Central	6 (6.0)	2 (3.8)	0 (0.0)	0 (0.0)
	West South Central	7 (7.0)	3 (5.8)	2 (3.2)	2 (6.2)
	Mountain	5 (5.0)	5 (9.6)	4 (6.4)	4 (12.5)
	Pacific	12 (12.0)	7 (13.5)	6 (9.5)	3 (9.4)
Practice types, *n* (%)	Single specialty group	63 (63.0)	32 (61.5)	45 (71.4)	20 (62.5)
	Multi-specialty Group	29 (29.0)	19 (36.5)	16 (25.4)	12 (37.5)
	Solo practitioner	3 (3.0)	0 (0.0)	0 (0.0)	0 (0.0)
	Other clinical settings	5 (5.0)	1 (1.9)	2 (3.2)	0 (0.0)
Months of follow-up, mean (SD)		69.0 (25.6)	74.9 (29.0)	71.3 (24.7)	75.6 (29.0)
Number of visits^b^, mean (SD)		3.5 (1.4)	3.3 (1.1)	3.4 (1.3)	3.3 (1.3)
BMI	*n* (%) patients with data	84 (84.0)	45 (86.5)	53 (84.1)	25 (78.1)
	Mean (SD)	30.2 (8.2)	31.5 (8.8)	29.9 (6.9)	30.5 (7.8)
Charlson comorbidity index^c^	*n* (%) patients with data	86 (86.0)	46 (88.5)	54 (85.7)	28 (87.5)
	Mean (SD)	1.7 (1.3)	1.8 (1.4)	1.9 (1.7)	2.4 (2.1)
Laboratory measures within 3 months prior to the index date					
ESR, mm/h	*n* (%) patients with data	59 (59.0)	33 (63.5)	35 (55.6)	15 (46.9)
	Mean (SD)	26.6 (23.2)	25.2 (22.1)	27.9 (23.4)	35.4 (30.5)
CRP, mg/dL	*n* (%) patients with data	59 (59.0)	30 (57.7)	39 (61.9)	17 (53.1)
	Mean (SD)	2.4 (4.5)	3.6 (9.9)	2.6 (5.1)	1.7 (3.0)
Prior bDMARD use^d^, *n* (%)	Adalimumab	2 (2.0)	1 (1.9)	2 (3.2)	1 (3.1)
	Etanercept	0 (0.0)	0 (0.0)	0 (0.0)	0 (0.0)
	Certolizumab	0 (0.0)	0 (0.0)	0 (0.0)	0 (0.0)
	Abatacept	1 (1.0)	1 (1.9)	1 (1.6)	0 (0.0)
	Rituximab	1 (1.0)	0 (0.0)	1 (1.6)	0 (0.0)
	Tocilizumab	1 (1.0)	1 (1.9)	1 (1.6)	1 (3.1)
Prior tsDMARD use^d^, *n* (%)	Tofacitinib	2 (2.0)	1 (1.9)	0 (0.0)	0 (0.0)
Average number of IV-infliximab prescriptions (within 6 months prior to the index date), mean (SD)		1.6 (0.7)	1.7 (0.8)	1.6 (0.7)	1.6 (0.8)
Days between last IV-infliximab prescription and index date, mean (SD)		43.5 (17.2)	42.9 (20.0)	44.6 (19.7)	45.8 (24.7)
Number of IV-infliximab prescriptions reported by *n* (%) patients within 6 months prior to the index date	1	43 (43.0)	23 (44.2)	30 (47.6)	16 (50.0)
	2	23 (23.0)	8 (15.4)	10 (15.9)	5 (15.6)
	3	19 (19.0)	12 (23.1)	15 (23.8)	6 (18.8)
	4	9 (9.0)	4 (7.7)	4 (6.4)	2 (6.2)
	> 4	6 (6.0)	5 (9.6)	4 (6.4)	3 (9.4)
Total IV-infliximab dose reported within 6 months prior to the index date (mg)	*n* (%) patients with data	49 (49.0)	26 (50.0)	31 (49.2)	15 (46.9)
	Mean (SD)	1737.2 (1065.5)	1685.8 (1289.3)	1723.5 (1038.3)	1661.4 (1302.6)
Concomitant rheumatologic medication use^e^, *n* (%)	Systemic glucocorticoids	61 (61.0)	37 (71.2)	39 (61.9)	22 (68.8)
	Methotrexate	42 (42.0)	27 (51.9)	25 (39.7)	12 (37.5)
	Hydroxychloroquine	17 (17.0)	9 (17.3)	9 (14.3)	3 (9.4)
	Leflunomide	19 (19.0)	9 (17.3)	11 (17.5)	6 (18.8)
	Azathioprine	4 (4.0)	3 (5.8)	5 (7.9)	4 (12.5)
	Sulfasalazine	8 (8.0)	3 (5.8)	6 (9.5)	2 (6.2)

^a^Others race: Native Hawaiian or other Pacific Islander, American Indian or Alaska Native, and multi-racial

^b^Number of visits with the rheumatologist in the 12 months prior to the index date

^c^Charlson comorbidity index was calculated during the 12 months prior to the index date

^d^Prior b/tsDMARD use defined as reported use 12 to 6 months prior to the index date

^e^Concomitant rheumatologic medication use was defined as any other reported drug use on or after the index date and while the patient was still receiving IV-golimumab

*bDMARD* biologic disease-modifying antirheumatic drug, *BMI* body mass index, *CDAI* Clinical Disease Activity Index, *CRP* C-reactive protein, *ESR* erythrocyte sedimentation rate, *IV* intravenous, *RA* rheumatoid arthritis, *RADA* rheumatoid arthritis disease activity, *RAPID3* Routine Assessment of Patient Index Data 3, *SD* standard deviation, *tsDMARD* targeted synthetic disease-modifying antirheumatic drug, *US* United States

### RA disease activity after switching from IV-infliximab to IV-golimumab

Among 52 patients with CDAI assessments, the mean (SD) CDAI score significantly improved from 21.3 (13.1) at baseline to 14.1 (9.8) during the 6-month follow-up period (*p* < 0.0001). During this timeframe, the proportion of patients with moderate/high disease activity decreased from 73 to 56%, and the proportion with low/remitted disease activity increased from 27 to 44% (*p* < 0.001). Significant improvements from baseline in CDAI scores were also observed during the 9-month follow-up (Table 2). Similar response patterns were observed with RAPID3 assessments during the 9-month follow-up (Online Resource 2).

**Table 2:** Mean CDAI scores and categories of disease activity for RA patients with persistent IV-golimumab use during the 6-month^a^ and 9-month^b^ follow-up periods

	*N* = 52			*N* = 32		
	Baseline	6-month follow-up^a^	*p* value	Baseline	9-month follow-up^b^	*p* value
Mean score (SD)	21.3 (13.1)	14.1 (9.8)	< 0.0001^c^	22.2 (13.9)	15.2 (14.1)	0.0002^c^
Disease activity categories^d^, *n* (%)						
Remission	0 (0.0)	2 (3.8)	< 0.001^e^	0 (0.0)	4 (12.5)	< 0.001^e^
Low	14 (26.9)	21 (40.4)		7 (21.9)	13 (40.6)	
Moderate	16 (30.8)	20 (38.5)		10 (31.2)	6 (18.8)	
High	22 (42.3)	9 (17.3)		15 (46.9)	9 (28.1)	

^a^Disease activity was assessed 6–9 months after the index date

^b^Disease activity was assessed 9–12 months after the index date

^c^Calculated using a paired *t*-test

^d^CDAI sums the number of swollen (0–28) and tender (0–28) joints and the rating of global disease activity (0–10) provided by the physician and patient. Scores range from 0 to 76; scores of ≤ 2.8, > 2.8–10.0, > 10.0–22.0, and > 22.0 represent remission, low, moderate, or high disease activity, respectively[16, 17]

^e^Calculated using a one-way repeated measures ANOVA test

*CDAI* clinical disease activity index, *IV* intravenous, *RA* rheumatoid arthritis, *SD* standard deviation

Sensitivity analyses of 81 patients with baseline and 6-month post-switch CDAI scores, regardless of dosing interval duration of IV-golimumab, similarly showed significant mean (SD) CDAI score improvement from 19.3 (12.4) at baseline to 13.6 (10.6) during the 6-month follow-up (*p* < 0.0001). Proportions of patients with moderate/high disease activity decreased from 70 to 49%, and with low/remitted disease activity increased from 30 to 51%, during the same timeframe (*p* < 0.001; Online Resource 3).

## Discussion

Treatment of RA with TNFi has proven effective after MTX/conventional synthetic DMARD combination treatment failure [19]. However, some patients treated with TNFi fail to achieve low disease activity, lose response over time, or experience adverse events, often prompting a dose increase, switch to a different TNFi, or switch to a biologic with a different mechanism of action. Potential neutralizing anti-drug antibody formation [10] and infusion reactions can reduce efficacy and limit therapeutic effect in ~ 25% of patients receiving IV-infliximab [11]. Such patients may benefit from switching to a fully human biologic, such as IV-golimumab, to control disease activity/improve drug tolerance. IV-golimumab has demonstrated long-term benefit in reducing RA signs and symptoms and improving physical function and is well-tolerated by patients [5]. IV-golimumab is less immunogenic than other TNFi [20]. Using data from the real-world RISE registry, we compared disease activity before and after direct switching from IV-infliximab to IV-golimumab in RA patients.

Patients switching from IV-infliximab to IV-golimumab generally had moderate-to-high disease activity at the time of IV-golimumab initiation. Of note, 44% received < 2 IV-infliximab doses during the 6 months prior to switching, potentially indicating under-treated disease, as maintenance treatment is usually given every 8 weeks. Reasons for the low number of doses were not captured (such as prior ineffectiveness, intolerance, insurance coverage). Significantly improved disease activity with ≥ 6 and ≥ 9 months of persistent IV-golimumab use post-switch may support the clinical benefit of this treatment approach among patients whose disease is inadequately controlled with IV-infliximab. Specifically, mean CDAI scores decreased by ~ 35% and ~ 30% at 6- and 9-month follow-ups, translating into almost 20% of patients shifting from moderate/high to low/remitted disease activity after switching to IV-golimumab. Statistical findings with the RAPID3 assessment were inconsistent between the 6- and 9-month follow-ups. A previous report indicates that, except for the patient global assessment, CDAI and RAPID3 components are not highly correlated [21]. The discordance of group-level data in this study is consistent with a previous IV-infliximab-to-IV-golimumab switch study [22] and is likely due to the exclusively patient-reported components that compose the RAPID3 score that may be conflated by other factors including anxiety, depression, and pain catastrophizing [17, 23, 24]. The CDAI, on the other hand, comprises a physician-reported scale plus a patient global score and total tender and swollen joint counts [16]; evidence points to tender and swollen joint counts as being the main CDAI score contributors [25].

### Limitations

The primary analysis included only patients who persistently used IV-golimumab during the 6- and 9-month follow-up periods, potentially introducing responder/selection bias. As a single-arm longitudinal study, confounding by indication is possible. These analyses are not comprehensive of patients’ reason(s) for switching from IV-infliximab, length of time on IV-infliximab, or dose escalation/de-escalation. Sample size was relatively small, and stratification of patients by full IV-infliximab history was therefore not possible. Follow-up was also relatively short for RA. A small number of RA patients also had ankylosing spondylitis, though the proportion was similar across follow-up and sensitivity analysis cohorts (≤ 5%). Despite these limitations, these real-world patients with moderate-to-high disease activity appeared to benefit from switching to IV-golimumab. Studies of longer duration will be needed to assess long-term effectiveness of IV-golimumab in these patients.

## Conclusion

Results of this study using RISE data suggest that IV-golimumab is effective in improving RA disease activity in a patient population switching directly from IV-infliximab, as measured by the CDAI, a standard disease activity index aligned with ACR and EULAR treat-to-target guidelines. Given the limited real-world data documenting efficacy of IV-infliximab-to-IV-golimumab switching, these results are clinically meaningful and may assist clinicians in providing patients with improved and sustained outcomes.

## Disclaimer

This analysis was supported by the American College of Rheumatology’s Rheumatology Informatics System for Effectiveness Registry. However, the views expressed represent those of the authors, not necessarily those of the American College of Rheumatology.

## Supplementary Information

Below is the link to the electronic supplementary material.

Supplementary file1 (DOCX 26 KB)

Supplementary file2 (DOCX 23 KB)

Supplementary file3 (DOCX 24 KB)

## Data Availability

The American College of Rheumatology (ACR) owns the data in the Rheumatology Informatics System for Effectiveness (RISE) registry, and University of California San Francisco, as a Data Analytic Center for the ACR, has access to the data for specific research projects, including this one, but is contractually obligated to not share this data, even in a de-identified state.
