# Supplementary material for: Improvement in disease activity among patients with rheumatoid arthritis who switched from intravenous infliximab to intravenous golimumab in the ACR RISE registry
Source: Clin Rheumatol. 2022 Mar 21;41(8):2319–27. doi: 10.1007/s10067-022-06116-z (PMC9287251; doi:10.1007/s10067-022-06116-z)
Supplement: Supplementary file 1 — Supplementary file1 (DOCX 26 KB) [file 10067_2022_6116_MOESM1_ESM.docx]

**Online Resource 1** Baseline characteristics of IV-golimumab RA patients of the sensitivity analysis who have any RADA score (CDAI or RAPID3) and who have a CDAI score only.

| Variables | | Patients with any RADA  (N=146) | Patients with CDAI  (N=81) |
| --- | --- | --- | --- |
| Age, years, mean (SD) | | 63.7 (12.2) | 62.2 (12.5) |
| Female, n (%) | | 118 (80.8) | 64 (79.0) |
| Race and ethnicity, n (%) | White (non-Hispanic) | 108 (74.0) | 63 (77.8) |
|  | Asian | 4 (2.7) | 2 (2.5) |
|  | Black or African American | 6 (4.1) | 4 (4.9) |
|  | Other^a^ | 7 (4.8) | 5 (6.2) |
|  | Missing | 13 (8.9) | 1 (1.2) |
|  | Hispanic/Latino | 8 (5.5) | 6 (7.4) |
| Insurance, n (%) | Medicare | 65 (44.5) | 34 (42.0) |
|  | Medicaid | 11 (7.5) | 8 (9.9) |
|  | Private | 50 (34.2) | 32 (39.5) |
|  | Other | 2 (1.4) | 2 (2.5) |
|  | Missing | 18 (12.3) | 5 (6.2) |
| US geographic divisions, n (%) | New England | 1 (0.7) | 0 (0.0) |
|  | Mid-Atlantic | 10 (6.8) | 6 (7.4) |
|  | East North Central | 29 (19.9) | 21 (25.9) |
|  | West North Central | 10 (6.8) | 2 (2.5) |
|  | South Atlantic | 45 (30.8) | 20 (24.7) |
|  | East South Central | 17 (11.6) | 8 (9.9) |
|  | West South Central | 12 (8.2) | 8 (9.9) |
|  | Mountain | 6 (4.1) | 6 (7.4) |
|  | Pacific | 16 (11.0 | 10 (12.4) |
| Practice types, n (%) | Single Specialty Group | 104 (71.2) | 54 (66.7) |
|  | Multi-Specialty Group | 34 (23.3) | 24 (29.6) |
|  | Solo Practitioner | 4 (2.7) | 1 (1.2) |
|  | Other Clinical Setting | 4 (2.7) | 2 (2.5) |
| Months of follow up time, mean (SD) | | 69.8 (27.5) | 73.0 (30.4) |
| Number of visits^b^, mean (SD) | | 3.2 (1.5) | 3.1 (1.5) |
| BMI | n (%) patients with data | 128 (87.7) | 72 (88.9) |
|  | Mean (SD) | 30.7 (8.5) | 31.2 (8.2) |
| Charlson comorbidity index^c^ | n (%) patients with data | 124 (84.9) | 73 (90.1) |
|  | Mean (SD) | 1.6 (1.2) | 1.7 (1.2) |
| Laboratory measures within 3 months pre-index date | | |  |
| ESR, mm/h | n (%) patients with data | 50 (34.2) | 32 (39.5) |
|  | Mean (SD) | 23.9 (17.9) | 25.3 (18.8) |
| CRP, mg/dL | n (%) patients with data | 81 (55.5) | 46 (56.8) |
|  | Mean (SD) | 2.6 (8.7) | 1.8 (3.1) |
| Prior bDMARD use^d^, N (%) | Adalimumab | 6 (4.1) | 4 (4.9) |
|  | Etanercept | 18 (12.3) | 8 (9.9) |
|  | Certolizumab | 14 (9.6) | 11 (13.6) |
|  | Abatacept | 3 (2.0) | 3 (3.7) |
|  | Rituximab | 2 (1.4) | 0 (0.0) |
|  | Tocilizumab | 3 (2.0) | 2 (2.5) |
| Prior tsDMARD use, n^d^ (%) | Tofacitinib | 3 (2.0) | 2 (2.5) |
| Average number of IV-infliximab prescriptions (within 6 months pre-index date), mean (SD) | | 1.6 (0.7) | 2.3 (1.1) |
| Number of IV-infliximab prescriptions reported by n (%) patients within 6 months pre-index date | 1 | 58 (39.7) | 34 (42.0) |
|  | 2 | 33 (22.6) | 15 (18.5) |
|  | 3 | 32 (21.9) | 17 (21.0) |
|  | 4 | 12 (8.2) | 6 (7.4) |
|  | >4 | 11 (7.5) | 9 (11.1) |
| Concomitant rheumatologic medication use^e^, n (%) | Systemic glucocorticoids | 117 (80.1) | 69 (85.2) |
|  | Methotrexate | 80 (54.8) | 45 (55.6) |
|  | Hydroxychloroquine | 24 (16.4) | 11 (13.6) |
|  | Leflunomide | 23 (15.8) | 12 (14.8) |
|  | Azathioprine | 5 (3.4) | 3 (3.7) |
|  | Sulfasalazine | 9 (6.2) | 3 (3.7) |

^a^Others race: Native Hawaiian or other Pacific Islander, American Indian or Alaska Native, and Multi-Racial.

^b^Number of visits with the rheumatologist in the 12 months prior to the index date.

^c^Charlson comorbidity index was calculated during 12 months prior to index date.

^d^Prior b/tsDMARD use defined as reported use 12 to 6 months prior to the index date.

^e^Concomitant rheumatologic medication use was defined as any other reported drug use on or after the index date and while the patient was still on IV-golimumab.

*bDMARD: biologic disease-modifying antirheumatic drug, BMI: body mass index, CDAI: clinical disease activity index, CRP: C-reactive protein, ESR: erythrocyte sedimentation rate, IV: intravenous, RA: rheumatoid arthritis, RADA: rheumatoid arthritis disease activity, SD: standard deviation, tsDMARD: targeted synthetic disease-modifying antirheumatic drug, US: United States*
