# Supplementary material for: Improvement in disease activity among patients with rheumatoid arthritis who switched from intravenous infliximab to intravenous golimumab in the ACR RISE registry
Source: Clin Rheumatol. 2022 Mar 21;41(8):2319–27. doi: 10.1007/s10067-022-06116-z (PMC9287251; doi:10.1007/s10067-022-06116-z)
Supplement: Supplementary file 2 — Supplementary file2 (DOCX 23 KB) [file 10067_2022_6116_MOESM2_ESM.docx]

**Online Resource 2** RAPID3 scores and categories of disease activity for RA patients with persistent IV-golimumab use during 6-month^a^ and 9-month^b^ follow-up periods.

|  | N=60 | | | N=45 | | |
| --- | --- | --- | --- | --- | --- | --- |
|  | Baseline | 6-month follow-up^a^ | P value | Baseline | 9-month follow-up^b^ | P value |
| Mean score (SD) | 11.4 (6.9) | 9.7 (7.3) | 0.007^c^ | 10.4 (6.6) | 10.6 (7.6) | 0.917^c^ |
| Disease activity categories^d^, n (%) | | | | | | |
| Remission | 6 (10.0) | 13 (21.7) | 0.004^e^ | 6 (13.3) | 9 (20.0) | 0.697^e^ |
| Low | 11 (18.3) | 16 (26.7) |  | 10 (22.2) | 7 (15.6) |  |
| Moderate | 14 (23.3) | 7 (11.7) |  | 12 (26.7) | 13 (28.9) |  |
| High | 29 (48.3) | 24 (40.0) |  | 17 (37.8) | 16 (35.6) |  |

^a^Disease activity was assessed 6-9 months after the index date.

^b^Disease activity was assessed 9-12 months after the index date.

^c^Calculated using a paired t-test.

^d^RAPID3 is a patient reported outcome measure without formal joint counts and comprises the Multidimensional Health Assessment Questionnaire patient self-report RA Core Data Set measures for physical function, pain, and patient global estimate (score range: 0-30). Scores range from 0-30; scores of 0-3.0, >3.0-6.0, >6.0-12.0, and >12.0 represent remission, low, moderate, or high disease activity, respectively.[17]

^e^Calculated using a one-way repeated measures ANOVA test.

*IV: intravenous, RA: rheumatoid arthritis, RAPID3: routine assessment of patient index data 3, SD: standard deviation*
