# Supplementary material for: Improvement in disease activity among patients with rheumatoid arthritis who switched from intravenous infliximab to intravenous golimumab in the ACR RISE registry
Source: Clin Rheumatol. 2022 Mar 21;41(8):2319–27. doi: 10.1007/s10067-022-06116-z (PMC9287251; doi:10.1007/s10067-022-06116-z)
Supplement: Supplementary file 3 — Supplementary file3 (DOCX 24 KB) [file 10067_2022_6116_MOESM3_ESM.docx]

**Online Resource 3** Mean CDAI scores and categories of disease activity for IV-golimumab RA patients of the sensitivity analysis.

|  | N=81 | | |
| --- | --- | --- | --- |
|  | Baseline | 6-month follow-up^a^ | P value |
| Mean score (SD) | 19.3 (12.4) | 13.6 (10.6) | <0.0001^b^ |
| Disease activity categories^c^, n (%) |  |  |  |
| Remission | 2 (2.5) | 4 (4.9) | <0.001^d^ |
| Low | 22 (27.2) | 37 (45.7) |  |
| Moderate | 26 (32.1) | 27 (33.3) |  |
| High | 31 (38.3) | 13 (16.0) |  |

^a^Disease activity was assessed 6-9 months after the index date.

^b^Calculated using a paired t-test.

^c^CDAI sums the number of swollen (0–28) and tender (0-28) joints and the rating of global disease activity (0–10) provided by the physician and patient. Scores range from 0-76; scores of ≤2.8, >2.8-10.0, >10.0-22.0, or >22.0 represent remission, low, moderate, or high disease activity, respectively [16,17].

^d^Calculated using a one-way repeated measures ANOVA test.

*CDAI: clinical disease activity index, IV: intravenous, RA: rheumatoid arthritis, SD: standard deviation*
